# Draft genome sequence of an XDR *Klebsiella pneumoniae* strain NGCE-700 isolated from human blood sample in Dhaka, Bangladesh

**DOI:** 10.1128/mra.01345-25

**Published:** 2026-02-09

**Authors:** Ismat Ruh Jahan, Syeda Naushin Tabassum, Aura Rahman, Fahad Khan, Abdus Sadique, Sakib Abrar Hossain, Arman Hossain, Jahidul Alam, Pritylata Mandal, Kamal Ahmed Chowdhury, Mohammad Mohsin, Maqsud Hossain

**Affiliations:** 1 Department of Biochemistry and Microbiology, North South University 54495, Dhaka, Bangladesh; 2 NSU Genome Research Institute (NGRI), North South University 54495, Dhaka, Bangladesh; 3 Department of Biochemistry, University of Regina 6846, Regina, Saskatchewan, Canada; 4 Department of Anaesthesiology, Sir Salimullah Medical College Hospital 469194, Dhaka, Bangladesh; 5 Critical Care Medicine, Sir Salimullah Medical College Hospital 469194, Dhaka, Bangladesh; 6 Popular Medical College and Hospital 469133, Dhaka, Bangladesh; Nanchang University, Nanchang, Jiangxi, China

**Keywords:** *Klebsiella pneumoniae*, extensively drug-resistant, bacteremia

## Abstract

We report the draft genome sequence of the extensively drug-resistant *Klebsiella pneumoniae* strain NGCE-700 isolated from a bacteremia patient in Bangladesh. The 5.53 Mb genome comprises nine contigs with N50 5.49 Mb and guanine or cytosine content of 57.3% and harbors multiple resistant genes and plasmids consistent with its XDR phenotype.

## ANNOUNCEMENT

*Klebsiella pneumoniae*, an opportunistic pathogen in the Enterobacteriaceae, is increasingly resistant to multiple antibiotics, leading to the emergence of MDR and XDR strains complicating infection control.

Here, we present the draft genome sequence of NGCE-700 generated using a hybrid assembly approach integrating reads from Illumina and Oxford Nanopore Technologies platforms.

The study was reviewed and approved by the Institutional Review Board/Ethics Review Committee (IRB/ERC) at North South University (CTRG-2020/OR-NSU/IRB/1205). NGCE-700 was isolated from the blood of a bacteremia patient from Dhaka Central International Medical College and Hospital, Dhaka, Bangladesh on 27 February 2019.

One milliliter of blood was enriched in 9 mL of Luria-Bertani (LB) broth (HiMedia, India) and incubated at 37°C for 18 h. A loopful of the enriched culture was streaked on MacConkey agar and incubated at 37°C overnight. Large, pink, mucoid colonies were obtained, and single colonies were subcultured.

Antimicrobial susceptibility testing (AST) was performed using the Kirby-Bauer disk diffusion method on Mueller-Hinton agar (MHA) following Clinical and Laboratory Standards Institute (CLSI) guidelines (M100, 2019 edition) at NSU Genome Research Institute. The strain was resistant to cephalosporin, polymyxin, tetracycline, penicillin, and macrolides while remaining susceptible to carbapenem and aminoglycosides, classifying it as XDR.

Genomic DNA was extracted from a single colony cultured overnight in LB broth at 37°C using the GeneJET Genomic DNA Purification Kit (ThermoFisher Scientific, USA). Whole-genome sequencing was performed using both Oxford Nanopore MinIon and Illumina MiSeq platforms. ONT libraries were prepared using the Rapid PCR Barcoding Kit (SQK-RPB004, Oxford Nanopore Technologies, UK) and sequenced using FLO-MIN106 (R9.4.1) flow cell on a MinION Mk1C platform. Guppy v5.0.11 was used for basecalling (Super Accuracy mode), followed by adapter trimming and quality filtering using Porechop v0.2.3 (1) and Filtlong v0.2.1 (2). Illumina libraries were prepared using the Illumina Nextera XT Kit (Illumina, Inc., USA) and sequenced on an llumina MiSeq platform using MiSeq Reagent Kit v2. Quality control and adapter trimming were performed using FastQC v0.11.9 (3) and Trimmomatic v0.39 (4). Hybrid-genome assembly was performed using Unicycler v0.5.1 (5), and scaffolding was performed using RagTag v.2.1.0 (6). All software was run using default parameters, unless otherwise noted. Genome completeness was evaluated using BUSCO v.6.0.0 (7). Taxonomic assignment was confirmed by average nucleotide identity (ANI) against GCA_002761315.1 (Table 1). Genome annotation was performed using Prokka 1.14.6 (8). Assembly quality and sequencing statistics are summarized in Table 1.

**TABLE 1:** Sequencing, assembly, and genome statistics of NGCE-700

Category	Parameter	Value
Nanopore sequencing	Reads	257,973
	Read N50	3,120
	Total bases	780.0 Mb
Illumina sequencing	Paired-end reads	2 × 150
	Read length	538,484
Assembly (hybrid)	Total genome size	5.52 Mb
	GC content	57.3
	Number of scaffolds	9
	Completeness (BUSCO)	98.2
	N50	5.49
Annotation	CDS	5,134
	tRNAs	77
	tmRNA	1
ANI	Identity to reference (GCA_002761315.1)	99.02

Resistance genes identified using ResFinder v.4.7.2 (9) and Comprehensive Antibiotic Resistance Database (CARD) v4.0.1 (10) proved to be consistent with the phenotypic AST. Detected genes included *oqxA* and *oqxB* (fluoroquinolone), *blaTEM-1ß* and *blaCTX-M-15* (first-generation cephalosporins), and *tet-A*, (tetracycline). Genes conferring resistance to aminoglycosides (*KpnE*, *KpnF*, *KpnG*, and *KpnH*) were also present.

Virulence genes identified using VFDB (updated on 14 Jan 2025) (11) via Abricate v1.0.1 (12) identified yersiniabactin biosynthetic protein responsible for iron acquisition and pathogenicity (13).Conjugative plasmids, *IncFII_1_pKP91* and *IncFIB(K)_1_Kpn3*, were identified using PlasmidFinder (14 Jan 2025) (14) via Abricate v1.0.1, both associated with antimicrobial resistance.

This genomic resource will enhance our understanding of the clinical *K. pneumoniae* strains circulating in healthcare settings in Bangladesh.

## Data Availability

The whole-genome sequence of *K. pneumoniae* NGCE-700 (hybrid assembly) has been deposited in the European Nucleotide Archive (ENA) with accession number GCA_977063125.2. Raw sequencing reads from Oxford Nanopore platform are available in NCBI under accession number SRR36246122, and Illumina reads are available under accession number SRR36849667, BioSample accession number SAMN53517085, and BioProject accession number PRJEB102989.
